# Pb(Mg_1/3_Nb_2/3_)-PbTiO_3_-Based Ultrasonic Transducer for Detecting Infiltrated Water in Pressurized Water Reactor Fuel Rods

**DOI:** 10.3390/s19122662

**Published:** 2019-06-13

**Authors:** Geonwoo Kim, Namkyoung Choi, Yong-Il Kim, Ki-Bok Kim

**Affiliations:** 1 Department of Science of Measurement, University of Science and Technology, 217, Gajeong-ro, Yuseong-gu, Daejeon 34113, Korea; Geonwoo.Kim@usda.gov (G.K.); gnokd@kriss.re.kr (N.C.); yikim@kriss.re.kr (Y.-I.K.); 2 Environmental Microbial and Food Safety Laboratory, Agricultural Research Service, U.S. Department of Agriculture, Powder Mill Rd. Bldg. 303, BARC-East, Beltsville, MD 20705, USA; 3 Center for Safety Measurement, Korea Research Institute of Standards and Science, 267, Gajeong-ro, Yuseong-gu, Daejeon 34113, Korea; 4 Center for Convergence Property Measurement, Korea Research Institute of Standards and Science, 267, Gajeong-ro, Yuseong-gu, Daejeon 34113, Korea

**Keywords:** nuclear power plants, pressurized water reactor fuel rods, ultrasonic transducer, PMN-PT, neutron irradiation, non-destructive evaluation

## Abstract

In this study, a high-sensitivity Pb(${\mathrm{Mg}}_{1/3}{\mathrm{Nb}}_{2/3}$)$O_{3}{-\mathrm{PbTiO}}_{3}$ (PMN-PT)-based ultrasonic transducer was developed for detecting defective pressurized water reactor (PWR) fuel rods. To apply the PMN-PT substance to nuclear power plant facilities, given the need to guarantee their robustness against radioactive materials, the effects of neutron irradiation on PMN-PT were investigated. As a result, the major piezo-electric constants of PMN-PT, such as the electrical impedance, dielectric constant, and piezo-electric charge constant, were found to vary within acceptable ranges. This means that the PMN-PT could be used as the piezo-electric material in the ultrasonic transducer for nuclear power plants. The newly developed ultrasonic transducer was simulated using a modified KLM model for the through-transmission method and fabricated under the same conditions as in the simulation. The through-transmitted waveforms of normal and defective PWR fuel rods were obtained and compared with simulated results in the time and frequency domains. The response waveforms of the newly developed ultrasonic transducer for pressurized water reactor (PWR) fuel rods showed good agreement with the simulation outcome and could clearly detect defective specimens with high sensitivity.

## 1. Introduction

A pressurized water reactor (PWR) fuel rod typically consists of sintered uranium dioxide pellets stacked into thin zircaloy tubes (~ 4–5 m), which are sealed by welding at both ends. The zircaloy tubes are the first protection against leakage of the dangerously radioactive fission products. If deterioration of a PWR fuel rod occurs in coolant, its fission products are leaked and the defective PWR fuel rod is filled with cooling water. Therefore, detecting the presence of infiltrated water in the PWR fuel rods, which indicates cladding failure and lack of mechanical integrity, is very important for the safety of PWRs. Ultrasonic transducers based on lead zirconate titanate (PZT) ceramics have been widely used in the non-destructive evaluation (NDE) of nuclear power plant (NPP) components as well as in various other industrial fields due to their high performance and ease of manufacture [1,2,3,4]. 

However, the PZT-based ultrasonic transducers have some limitations for application to the high-attenuation materials used in NPPs because of its low piezo-electric constant [5,6]. In contrast, Pb (${\mathrm{Mg}}_{1/3}{\mathrm{Nb}}_{2/3}$)$O_{3}{-\mathrm{PbTiO}}_{3}$ (PMN-PT) single crystal is currently considered one of the most important among the emerging piezo-electric materials for high-sensitivity ultrasonic transducers. This is because of its superior piezo-electric constants, including exceptional piezo-electric charge constant ($d_{33}\geq$ 1500 pC/N), high electro-mechanical coupling factor ($k_{33}\;\sim \;$0.9), and superior dielectric constant ($\varepsilon_{r}\;\sim$ 5000) [5,6,7,8,9,10]. In addition, these piezo-electric constants can provide good sensitivity and bandwidth for ultrasonic imaging techniques [10,11,12]. In spite of these advantages, PMN-PT is not widely used for NDE of NPP applications because there has been little conformance verification on its radiation and high-temperature resistance. Much research about the effects of various kinds of irradiation on ferro-, pyro-, and piezo-electric elements have been reported [13,14,15]. However, to the best of our knowledge, only a few studies on the effects of radiation on PMN-PT have been conducted [7,16]. In our previous study, we investigated the effects of neutron irradiation on PMN-28%PT for NPP applications [16]. Increasing the neutron radiation dose to 160 Nm/g (Mrad) changes the major piezo-electric constants, which can affect the overall performance of ultrasonic transducers. For this reason, a number of qualities such as the dielectric constant $\varepsilon_{r}$, piezo-electric coefficient $d_{33}$, electrical impedance |Z|, and electrical resistance (ohm, Ω) were measured and analyzed. In addition, X-ray diffraction (XRD) was used to analyze the changes of the piezo-electric constant values. Angadi et al. reported the radiation response of PMN-PT to high-energy heavy ions (50 MeV Li^3+^, fluence 1 × 10^13–1^ × 10^14^ ions/cm^2^), in terms of its structural, dielectric, and piezo-electric constants at high temperature (180 °C) [7]. The current NDT methods to evaluate the extent of failure of in-service or post irradiated fuel rods are eddy current testing (ECT) and ultrasonic testing (UT) [17]. UT testing for in-service NPPs is typically conducted by its maintenance periodic testing activities after reactor shutting down and the temperature of coolant system is cooled down below 93 °C [1,18,19]. The two experimental studies clearly demonstrated that the piezo-electric constant and crystallinity of the material showed only slight changes (within acceptable ranges) under harsh environments and that variation of the major piezo-electric constants hardly affected the overall piezo-electric performance of PMN-PT. 

Therefore, we developed a PMN-PT-based high-sensitivity 5 MHz ultrasonic transducer for detecting defective PWR fuel rods of in-service PWRs. To accomplish this, we designed the ultrasonic transducer and simulated its response waveforms. For the ultrasonic transducer design and simulation, various models based on equivalent circuits have been developed such as the Mason, Redwood, and KLM models [20,21,22]. Among these models, the KLM model was selected for this study due to its simple acoustic transmission nodes and transfer functions that operate in a more physically intuitive manner than in the others [23,24,25,26]. The KLM model can simulate the thickness-vibration mode of a piezo-electric material, from which its ultrasonic pulse-echo response signals can be obtained. However, because the technique for detection of the permeated water is the through-transmission method, in this study, we used a modified KLM model developed in a previous study for the ultrasonic through-transmission method [25]. Then, the through-transmitted waveforms of defective PWR fuel rods were simulated. To verify the outcome, a prototype ultrasonic transducer was fabricated, and the experimental set up duplicated the same conditions used in the simulation. Then, the through-transmitted waveforms of normal and defective PWR fuel rods were obtained in time and frequency domains and compared with the simulation results.

## 2. Neutron Irradiation Effects on PMN-28%PT

**Figure 1:**
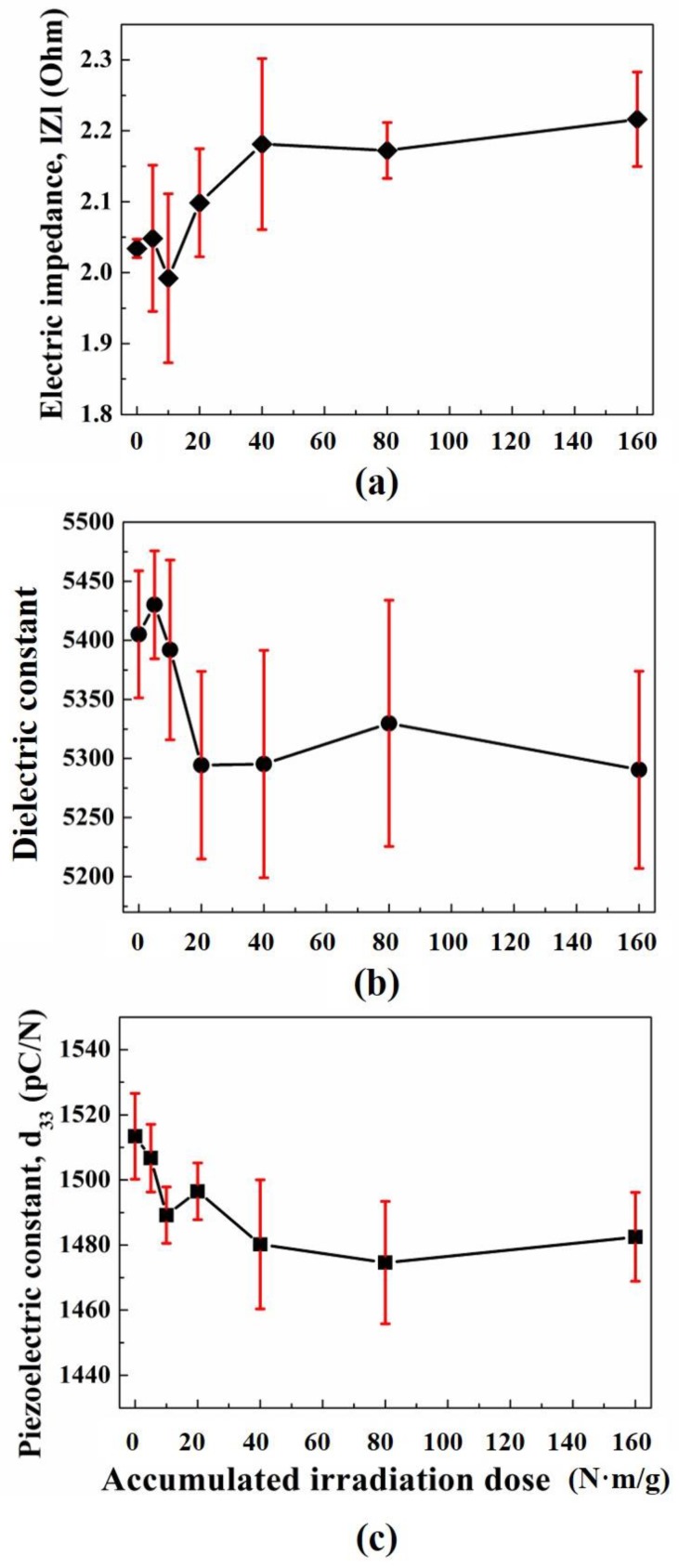
Results of the neutron irradiation effects on three PMN-28%PT samples: (**a**) Electrical impedance, |Z|; (**b**) dielectric constant, $\varepsilon_{r}$; (**c**) piezo-electric charge constant, $d_{33}$.

In our previous study, according to neutron irradiation dose on PMN-28%PT (Ibulephotonics Co., Ltd., Republic of Korea), changes of its electrical impedance, |Z|, dielectric constant, $\varepsilon_{r}$, and piezoelectric charge constant, $d_{33}$ were measured by an impedance and gain-phase analyzer (HP 4194A, Hewllet Packard Co., Ltd., Palo Alto, CA, USA), LCR meter (4263B LCR meter, Agilent Co., Ltd, Santa Clara, CA, USA), and $d_{33}$-meter (ZJ-6B PIEZO $d_{33}$/$d_{31}$ meter, Institute of Acoustics Chinese Academy of Sciences, Beijing, China), respectively, and its results were analyzed by X-ray diffractometry (D/Max 2200, Rigaku Co., Ltd., Tokyo, Japan). Also, obtained XRD pattern data were sorted by search–match method [16]. The number of PMN-28%PT samples was three and those were irradiated by Cf-252 neutron source from 0 to 160 Nm/g (Mrad) at a neutron dose rate of 50 Nm/g/h (Mrad/h) at room temperature. The center frequency and diameter of PMN-28%PT samples were 1 MHz and 1.8 mm. Figure 1 shows the results of the neutron irradiation effects on three PMN-28%PT samples.

**Figure 2:**
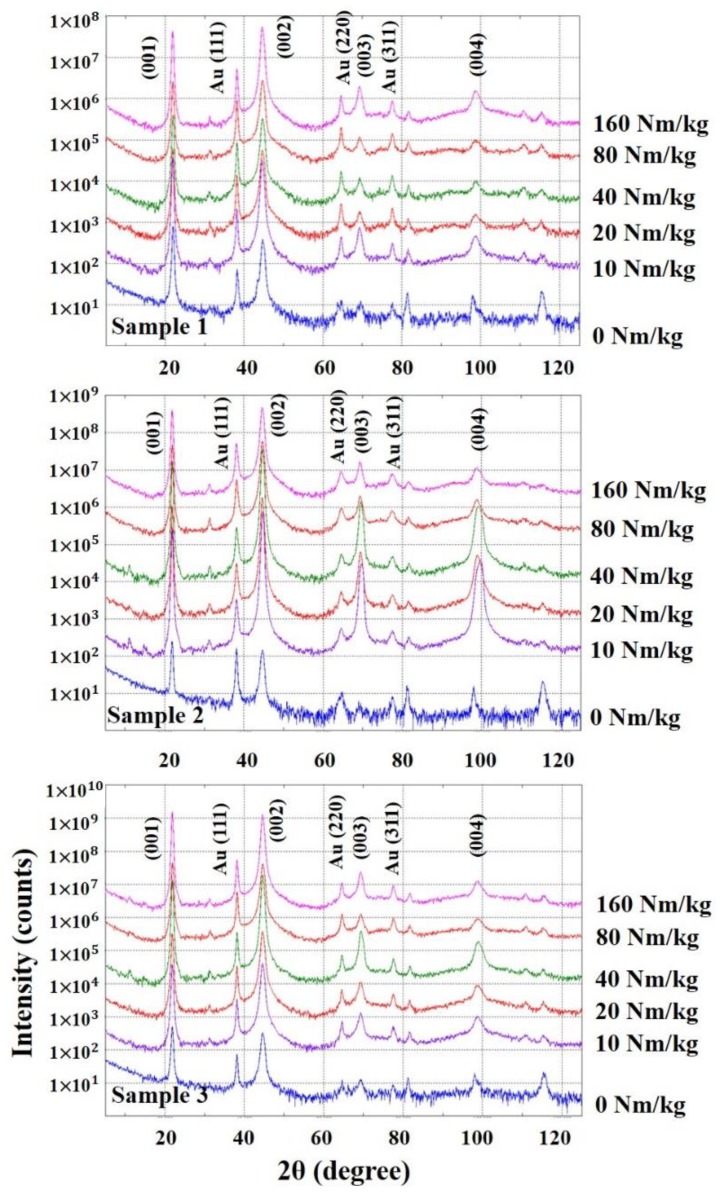
The intensity variation of three PMN-28%PT XRD patterns at (001) plane according to irradiation does levels.

In Figure 1, the black lines are the average of three samples and red vertical lines represented the 95% confidence intervals. In Figure 1a, the electrical impedance increased up to 40 Nm/g dose and after 40 Nm/g, it maintained constant values. In Figure 1b, permittivity slightly increased and decreased rapidly up to 20 Nm/g. Then, it represented uniform values. In Figure 1c, $d_{33}$ decreased up to 40 Nm/g and maintained uniform values up to 160 Nm/g. To analyze the causes of those property changes, its XRD pattern analysis was performed. The measurement conditions of the XRD experiment is summarized in Table 1, and Figure 2 shows obtained XRD patterns of three PMN-28%PT samples.

**Figure 3:**
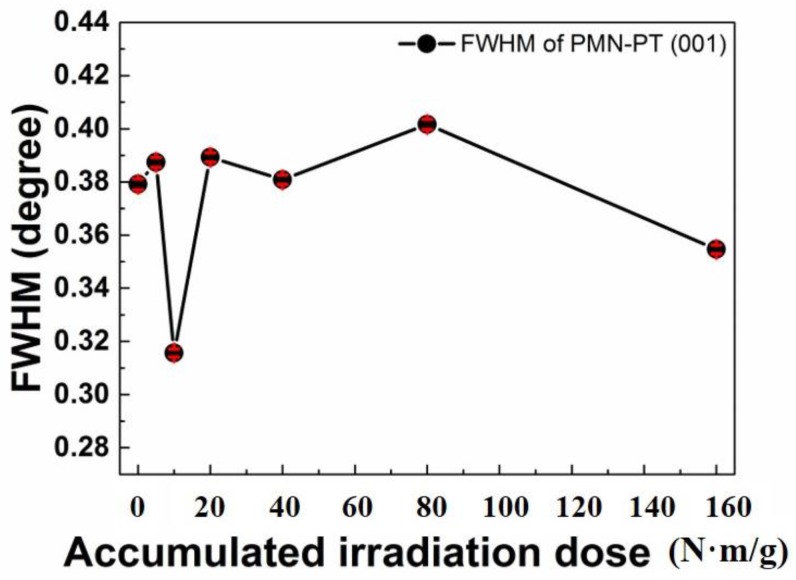
Variation of full width at half maximum (FWHM) at (001) plane of PMN-28%PT in accordance with the accumulated neutron irradiation does.

In Figure 2, XRD pattern of (001) plane of PMN-28%PT and Au (111) peak appeared very high. (111) peak is the electrode (Au) of PMN-28%PT. From the obtained XRD data for analyzing the formation of the PMN-28%PT samples in single phase, the radioactive irradiation affected the material structure of PMN-28%PT. The crystal structures of PMN-28%PT, which can be described by [(1-x)Pb(Mg_1/3_Nb_2/3_)O_3_-xPbTiO_3_] are rhombohedral, monoclinic, tetragonal, and cubic phase at room temperature in accordance with the amount of PbTiO_3_ content [16]. From Figure 2, the crystal structure of PMN-28%PT samples showed tetragonal phase. The polarization of PMN-28%PT, which has tetragonal phase, can be influenced by the degree of off-center of atoms occupied the (00*l*) reflection. In addition, gradual increase of X-ray diffracted intensities for (002), (003), and (004) reflections means the increase of reflection plane density due to atoms occupied the (00*l*) toward the center position. Consequently, the degradation of $d_{33}$ and dielectric constant was affected by this movement of atoms. In the view point of qualitative analysis, its phase was not changed. However, the full width at half maximum (FWHM), which represents its crystallinity, irregularly varied according to the irradiation does as shown in Figure 3. 

In Figure 3, the variation of the FWHM diffraction peak reflects the change of its crystallinity with an increase in the radiation dose. Also, the irregularity of its crystallinity is one of the important major causes that develop defect density such as sample dislocation. This phenomenon can cause the permanent dipole moment decreases in the PMN-28%PT crystal structure. From the above analysis, we concluded that the variation of major piezo-electric constants can be caused by neutron irradiation dose [16]. Although there was slight decrease in the PMN-28%PT crystallinity according to increasing neutron irradiation dose, the major piezo-electric constants, which can decide the ultrasonic transducer performance, such as electrical impedance, dielectric constant, and piezoelectric charge constant, varied within acceptable ranges as shown in Figure 1. Therefore, the PMN-28%PT single crystal could be a promising piezo-electric material for NPPs application.

## 3. Ultrasonic Transducer Design

### 3.1. Structure of Ultrasonic System and Transducer

**Figure 4:**
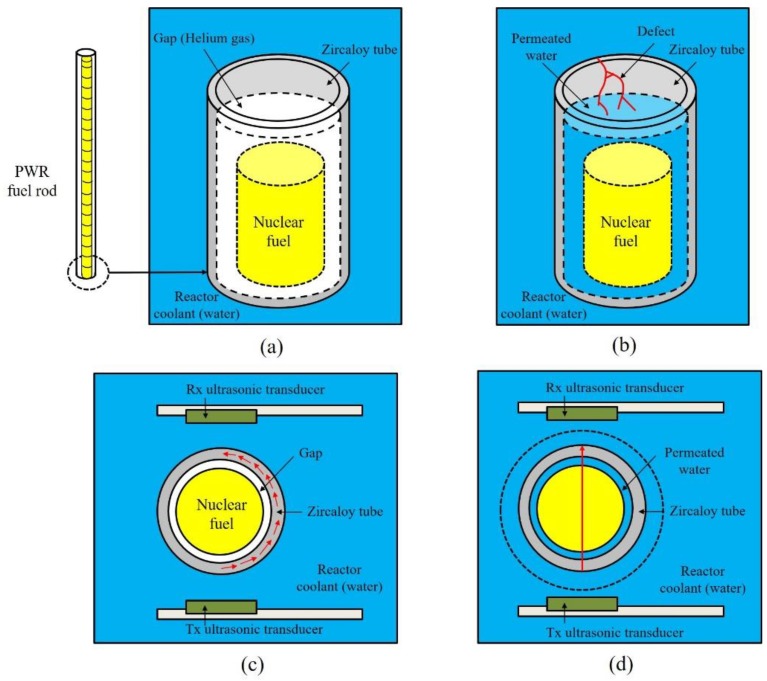
Conceptual diagram of sound and defective pressurized water reactor (PWR) fuel rods and its ultrasonic inspection: (**a**) Sound PWR fuel rod; (**b**) defective PWR fuel rod with permeated water from reactor coolant; (**c**) ultrasonic inspection of sound PWR fuel rod and its ultrasonic path, which is trapped in PWR fuel rod; (**d**) ultrasonic inspection of defective PWR fuel rod and through-transmitted ultrasonic path.

The ultrasonic testing system for detecting defective PWR fuel rods consisted of transmitting (Tx) and receiving (Rx) ultrasonic transducers, a test specimen, an oscilloscope, a pulse generator, and a receiver. Unlike a pulse-echo method, which uses one ultrasonic transducer, the through-transmission method utilizes both Tx and Rx ultrasonic transducers. The conceptual diagram of a device inspecting PWR fuel rods is shown in Figure 4. 

Figure 4a,b shows the sound and defective PWR fuel rods. Uranium powder baked into cylindrical yellow pellets were stacked into a thin zircaloy tube. The outer diameter and thickness of PWR fuel rod is 10 mm and 1 mm, respectively. There is a gap between the tube and the pellets, and the gap is filled with helium gas to improve heat transport from the nuclear fuel to the reactor coolant [2,3,4]. However, in the case of defective fuel rod, the cooling water can be permeated through the zircaloy tube and the gap is filled with the water instead of the gas. When PWR fuel rods are inspected, the main way to receive the elastic waves generated by the Tx ultrasonic transducer is via the tube, as described in Figure 4c, and the distance between both ultrasonic transducers is about 12 mm. If defects occur, cooling water fills the gas gap and the ultrasonic waves are directly transmitted to the Rx ultrasonic transducer, as represented in part Figure 4d. Therefore, the NDE of the PWR fuel rods is conducted by analyzing the through-transmitted ultrasonic signals propagated within the zircaloy tubes.

**Figure 5:**
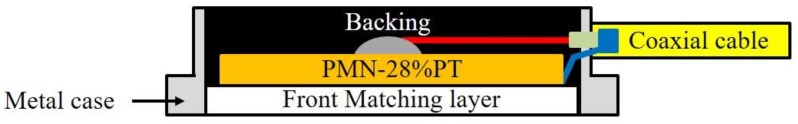
Structure of the PMN-28%PT ultrasonic transducer developed for non-destructive evaluation (NDE) of PWR fuel rods.

The structure of the ultrasonic transducer includes the piezo-electric element, front matching layers, metal cases, and backing materials. The piezo-electric material transforms the acoustic energy into electrical signals, and vice versa. The backing materials act like a mechanical damper that absorbs the ultrasonic waves generated on the back side of the piezo-electric element. It also determines the bandwidth of the response waveforms by controlling their acoustic impedance [25,26,27,28]. The front matching layer is located between the piezo-electric element and a test specimen and serves as an acoustic impedance matching filter to minimize loss of the acoustic energy and to control the phase of the transmitted ultrasonic signals [5,6,25,26,27,28]. The structure of the PMN-28%PT ultrasonic transducer developed for NDE of PWR fuel rods is shown in Figure 5.

To serve as the acoustic matching filter, the acoustic impedance of the front matching layer must be derived, and its ideal acoustic impedance is defined in Equation (1) [27,28].

(1) $$Z_{2}\;=\;\sqrt{Z_{1}\cdot Z_{3}},$$

where *Z*_1_ and *Z*_3_ are the acoustic impedance of the piezo-electric material and medium (a test specimen), respectively. Because the PWR fuel rods are in the coolant, the acoustic impedance of the medium was assumed to be that of water (1.5 × 10^6^ kg/m^2^s). The calculated acoustic impedance for the front matching layer was 6.6 × 10^6^ kg/m^2^s and poly methyl methacrylate (4 × 10^6^ kg/m^2^s) was selected because of its ease of mechanical working and low acoustic attenuation coefficient. In addition, the appropriate thickness of the front matching layer is one quarter of its wavelength at the operating frequency of the ultrasonic transducer, and the calculated thickness of the poly methyl methacrylate was about 0.1 mm [27,28]. In addition, to determine the acoustic impedance of the backing material, backing materials with various acoustic impedance were simulated using the KLM model. From this, 8 × 10^6^ kg/m^2^s was selected as the acoustic impedance of the ultrasonic transducer, with about 60% bandwidth as in our previous studies [5,6,25,26].

### 3.2. KLM Model Modified for the Through-Transmission Method

In our previous study, according to the neutron irradiation dose on PMN-28%PT, changes of its electrical impedance, |*Z*|, the dielectric constant, $\varepsilon_{r}$, and the piezo-electric charge constant, $d_{33}$ were measured and analyzed because they are the crucial factors that affect the sensitivity and bandwidth of ultrasonic transducers [16,27,28,29]. The KLM modeling codes for ultrasonic through-transmission method are included in Supplementary file 1. To improve the electrical transmission loss and to control the bandwidth, the electrical impedance of piezo-electric materials is used to design the electric tuning circuit, which is located between the ultrasonic transducer and connected ultrasonic system. In particular, the dielectric constant and piezo-electric charge constant can directly affect the sensitivity of an ultrasonic transducer [20,21,22]. From XRD results, the major properties of PMN-28%PT are known to change its crystallinity with increasing neutron dose (from 0 to 16 Mrad). The variation was within an acceptable range (about ±5%) [16]. Therefore, we concluded that PMN-28%PT could be applied for NDE in NPPs. The material properties of the PMN-28%PT used in this study are summarized in Table 1 and the same properties were also used for the simulation.

**Figure 6:**
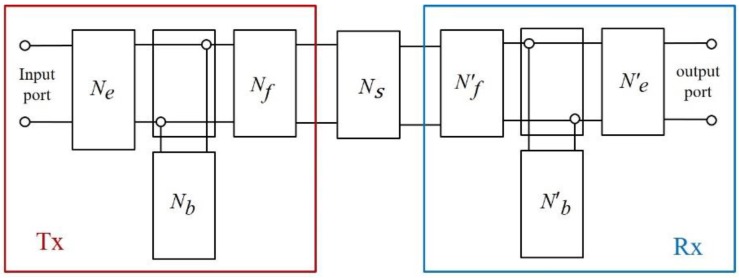
Schematic diagram of the modified KLM model for the through-transmission method.

In Table 2, the size of the PMN-28%PT was 3 × 3 mm because it should be smaller than the diameter of the PWR fuel rods (10 mm) to prevent the acoustic energy generated by the Tx ultrasonic transducer from being directly transmitted to the Rx ultrasonic transducer. The dielectric constant and piezo-electric constant were measured using an LCR meter (4263B LCR meter, Agilent Co., Ltd., Santa Clara, CA, USA) and $d_{33}$-meter (ZJ-6B PIEZO $d_{33}$/$d_{31}$ meter (Institute of Acoustics Chinese Academy of Sciences, Beijing, China), respectively. The acoustic impedance of the PMN-28%PT, including its density and sound speed, was determined using XRD diffraction analysis and the ultrasonic pulse-echo method. The curie temperature of PMN-28%PT is 160 °C and the major piezo-electric constants such as dielectric constant and $d_{33}$, which decide the sensitivity of ultrasonic transducer showed acceptable ranges in other studies [7,10]. To simulate the through-transmitted waveforms, we developed the modified KLM model: A schematic including a test specimen and ultrasonic transducers (Tx and Rx) is shown in Figure 6 [25]. 

**Figure 7:**
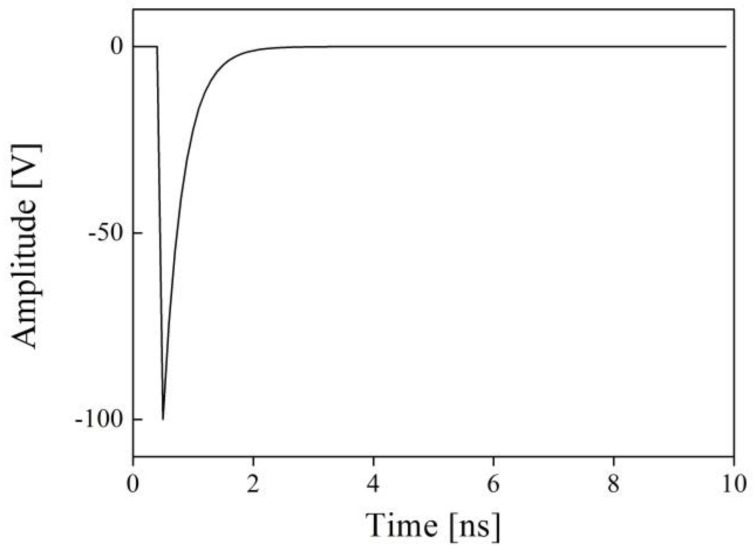
Simulated excitation pulse used in the KLM model modified for the through-transmission method.

In Figure 6, the components of the KLM model modified for the through-transmission method consist of equivalent circuits of the electro-mechanical coupling circuit *N_e_*, backing material *N_b_*, front matching layer *N_f_*, and the medium *Ns* (a test specimen). The Tx ultrasonic transducer is excited by an excitation signal at the input port and the signal is converted to an ultrasonic wave described as frequency-domain matrices by an electro-mechanical coupling circuit. Then, the ultrasonic signal is tuned by the backing and the front matching layer and transmitted to the Rx ultrasonic transducer through the medium. Each component of the modified KLM model is described as a series of transfer matrices and the final through-transmitted outcome is obtained by a total transmission function in frequency domain. Finally, the result is transformed in time domain using inverse fast Fourier transform (IFFT) [22,23,24,25]. The excitation pulse had to be determined and a spike pulse was selected due to its broadband characteristics. The broadband type ultrasonic transducers have been widely used for various NDE applications because they can produce high-resolution characteristics for measuring thickness, sizing of defects, ultrasonic imaging techniques, and so on [27,28]. Figure 7 represents the excitation pulse used in this study.

In Figure 7, the delay and rise time of the excitation pulse was 0.2 and 2 ns, respectively, and the pulse voltage and relative bandwidth of the excitation pulse were −100 V and 150 MHz at −3 dB. The modified KLM simulation conditions are summarized in Table 3. The raw data of excitation pulse is included in Supplementary file 2.

In Table 3, the acoustic impedance of the front matching layer was derived using Equation (1) and the acoustic impedance of the bonding material was the same as that of the epoxy resin. Based on these design conditions, the through-transmitted waveforms were simulated and the prototype ultrasonic transducer for NDE of PWR fuel rods was fabricated.

## 4. Results and Discussion

### 4.1. Prototype Ultrasonic Transducer for NDE of PWR Fuel Rods

**Figure 8:**
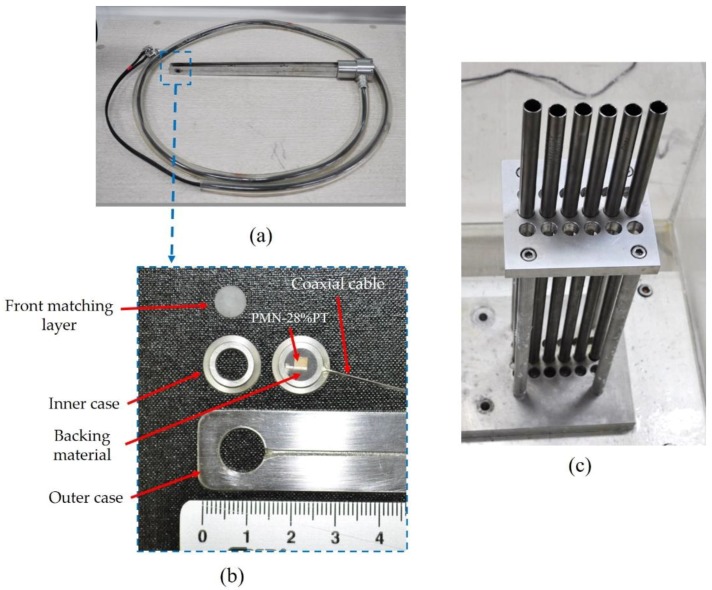
Newly developed PMN-28%PT-based ultrasonic transducer for NDE of PWR fuel rods: (**a**) Prototype ultrasonic transducer; (**b**) magnified image of ultrasonic transducer and its components; (**c**) PWR fuel rod assembly samples.

The prototype ultrasonic transducer for NDE of PWR fuel rods was fabricated under the same conditions used in the modified KLM simulation and is represented in Figure 8.

In Figure 8a,b, it can be seen that the newly developed ultrasonic transducer is composed of thin coaxial cable (≤0.3 mm), poly methyl methacrylate (a front matching layer), backing material, long coaxial cables (1 m, 50 Ω), and the inner and outer cases (stainless steel). The outer shield and center conductor of the coaxial cable was connected to both surfaces of the PMN-28%PT by a flat silver wire (25 μm, California Fine Wire Company, USA) with conductive metal epoxy (TC-2707, 3M, USA). The inner case was also bonded to the front matching layer and to the backing material by epoxy resin (Araldite GY509, Huntsman, USA) as shown in Figure 2. In this study, the radioactive resistance test for the backing material was not performed. However, we applied the backing material in this study because it has been used to our previous research works for the last 10-year period [5,25,26] and to the best of our knowledge, it was not easy to find proper backing materials with radioactive resistance for NPPs applications. Therefore, its radioactive resistance test will be also performed as a future work. The outer case was connected to the inner case assembly. The ultrasonic transducer for NDE of PWR fuel rods is typically designed to be inserted among the PWR fuel rods. These are arranged in a greedy assembly to prevent nuclear fuel vibration and movement in all directions, as shown in Figure 8c. In Figure 8a,c, the thickness and the center-to-center distance among the PWR fuel rods was 1 mm and 14 mm, respectively. Therefore, the distance between Tx and Rx transducers was 12 mm. 

To fabricate the attenuating material for the backing, 25 μm tungsten powder (W006016, Good Fellow, England) was mixed with epoxy resin. The attenuating material needs high density and ultrasonic propagation speed, and strong scattering effect because its acoustic impedance is the function of the density and sound speed [28]. Tungsten satisfies these material characteristics (19,300 ${\mathrm{kg}/m}^{3}$ for the density and 5180 m/s for the sound velocity). Moreover, its powder particles can effectively scatter the ultrasonic waves as well as absorb the mechanical vibration generated from the backside of the PMN-28%PT. In the middle of stirring the mixture, a lot of air bubbles can flow into the backing material. These should be removed because they decrease the acoustic impedance and the scattering effects. Therefore, a vacuum desiccator (VDC-31, Jeio Tech Co., Ltd., Daejeon, Korea) was used to eliminate the voids. Because the viscosity of the adhesive material should be low to mix with tungsten powder, the araldite epoxy resin was selected (about 5 Pas). Finally, the through-transmitted waveforms of normal and defective specimens were acquired using the newly developed ultrasonic transducer and compared with the simulation outcome.

### 4.2. Through-Transmitted Waveforms and Comparison with the Simulation Resutls

**Figure 9:**
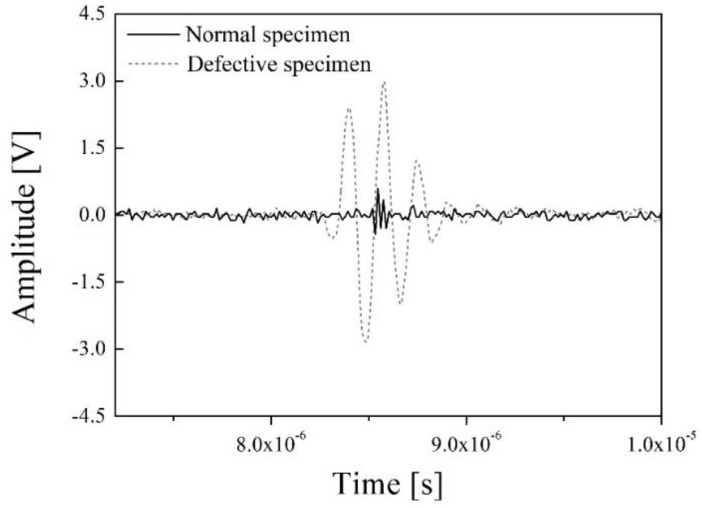
Through-transmitted response waveforms of the normal and defective specimens.

The experimental set-up of the through-transmission method includes the newly developed ultrasonic transducer for NDE of PWR fuel rods, an oscilloscope (Wave Runner 640ZI, Lecroy, Chestnut Ridge, NY, USA), a pulse generator and receiver (HIS2, Krautkramer, Tokyo, Japan), and the defective and normal specimens. The excitation signal of the pulse generator was a spike pulse, and its amplitude and bandwidth were −100 V and 150 MHz at −3 dB and the same excitation pulse was simulated in Figure 4. As mentioned above, the PWR fuel rods are filled with sintered uranium dioxide pellets and they emit various radioactive fission products. Therefore, in this study, a PWR fuel rod specimen with sintered uranium dioxide pellets was not used for safety reasons. To verify the through-transmitted response waveforms of the designed ultrasonic transducer, we obtained the through-transmitted response signals of an empty PWR fuel rod (a normal specimen) and a water-filled PWR fuel rod (a defective specimen). Although the PWR fuel rod was not packed with the uranium pellets, it was enough to compare the experimental outcome with the simulation results, because the conditions of the experimental set-up and simulation were the same. Figure 9 represents the through-transmitted waveforms of both specimens. 

**Figure 10:**
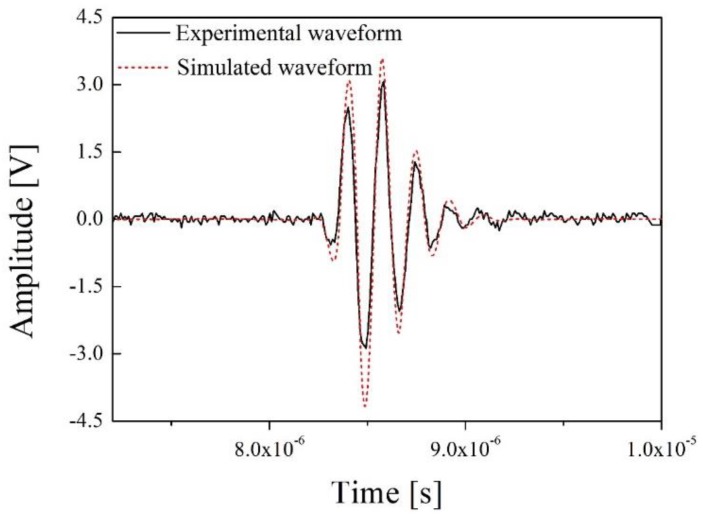
Through-transmitted waveforms of the defective specimen and its simulation result.

In Figure 9, the black line and gray dashes are the through-transmitted response waveforms of the normal specimen and the defective specimen, respectively. The peak-to-peak amplitudes of the normal specimen and defective specimen were about 0.9 and 5.7 V. As noted above, theoretically, the main path along which the ultrasonic energy is transmitted in the normal specimen is through a zircaloy tube described in Figure 4a. However, most of the transmitted signals can be trapped, reflected, and refracted in the tube due to its complex internal structure, cylindrical external shape, and relatively larger size than the sensing parts of the ultrasonic transducers, as shown in Figure 8a,b. In addition, because the acoustic impedance of each of the filling materials within the zircaloy tube is very different, the total acoustic energy transmission efficiency is very low. Therefore, those effects cause a large amount of acoustic energy loss during acoustic energy transfer. Although the normal PWR fuel rods are filled with the sintered uranium dioxide pellets, similar results should be derived in this case because the difference in the acoustic impedance of helium gas (0.00017 × 10^6^ kg/m^2^s) and a zircaloy tube (42.6 × 10^6^ kg/m^2^s) is very large. Thus, the transmitted acoustic waves are trapped in the tube due to the difference of acoustic impedance between the two materials and are reflected in various directions on the surface of the normal specimen. Therefore, the amplitude of the transmitted ultrasonic waveforms of the normal PWR fuel rods was very low or could be almost zero. From the above experimental results and analysis, simulation of the normal specimen was not conducted because its amplitude was too low to measure. In addition, the simulated through-transmitted response time of the defective PWR fuel rod was approximately 8.6 × 10^−6^, which was considering the thickness of fuel rod (2 mm), permeated water (10 mm), and the distance between Tx and Rx ultrasonic transducers. However, we could not obtain the through-transmitted response time of the normal PWR fuel rod. In the case of ultrasonic inspection for normal PWR fuel rods, shown in Figure 1 and Figure 5, three-dimensional cylindrical structures should be considered, and its acoustic energy would be trapped in round-shaped ring due to the great acoustic difference between helium has gap and PWR fuel rod. However, the KLM simulation cannot perform the three-dimensional acoustic wave propagation analysis and only two-dimensional analysis can be only performed. Therefore, acoustic energy is reflected on the helium gas layer and the simulated amplitude of normal PWR fuel rod was almost zero. To acquire more accurate results for the normal PWR fuel rods, we concluded that the additional study based on infinite element analysis is needed. Figure 10 shows the through-transmitted signals of the defective specimen and its simulation outcome.

**Figure 11:**
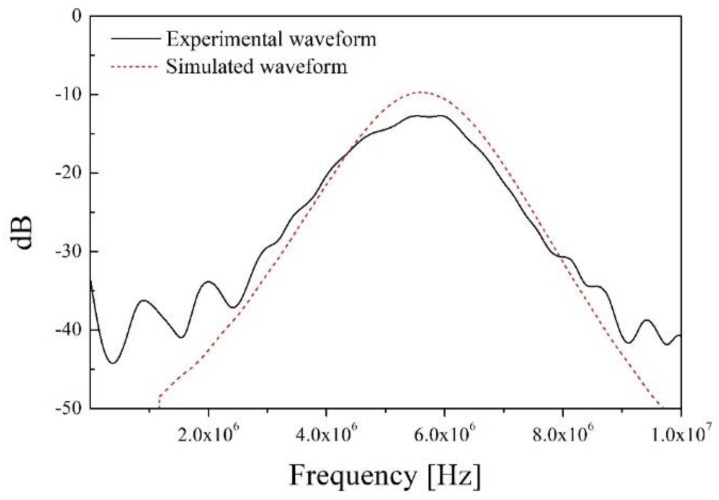
Frequency spectrum of through-transmitted waveforms of the defective specimen.

In Figure 10, the black line is the experimental through-transmitted waveform of the defective specimen and the red dashes describe its simulated waveform. The peak-to-peak amplitude of the simulated waveform is approximately 7.6 V, which is about 0.4 dB higher than its experimental result. In the simulation, the acoustic impedance of the defective specimen included only those of the tube and water. However, in the real world, the response waveform is affected by the spherical geometry of the zircaloy tube and it can cause acoustic energy loss and attenuation. Moreover, the electrical tuning needed to match the electrical impedance of the ultrasonic transducer to the ultrasonic system, including the oscilloscope and pulse generator, was not performed because the radioactive resistance of the electrical circuit and related effects were not investigated in this study. In addition, all electrical impedance in the simulation is ideally matched to 50 Ω. However, in our previous study, the electrical impedance of PMN-28%PT was changed with increase in the neutron radiation dose [16]. Therefore, it was not easy to design an optimal electrical tuning circuit due to its radioactivity dependence. In spite of the difficulties, the simulation results showed good agreement with the experimental results, only excepting the amplitude. Figure 11 represents the frequency spectrum (fast Fourier transform, FFT) of the two through-transmitted waveforms.

In Figure 11, the center frequency and bandwidth at −6 dB of two waveforms are almost same (about 5.5 MHz for the center frequency and about 40% for the bandwidth). In our previous study, backing material of about 8 × 10^6^ kg/m^2^s was simulated and a PZT-based ultrasonic transducer with approximately 60% bandwidth was developed [25]. However, in this study, the bandwidth decreased to about 40% from 60% because of the high piezo-electric charge constant, *d*_33_. PMN-PT produced high-sensitivity response signals due to its exceptional piezo-electric coefficient. However, its pulse duration was also increased because of its strong electro-mechanical conversion efficiency. The measured piezo-electric charge of the PMN-28%PT used was approximately 1515 pC/N, and this value is about three times higher than that of the normal PZTs widely used in the NDE field. From this point of view, the bandwidth is in inverse proportion to the piezo-electric charge constant, and the bandwidth of the newly developed ultrasonic transducer was also decreased. However, this is not a serious problem for detecting the infiltrated water in PWR rods. As shown in Figure 9, the clear difference between both through-transmitted waveforms of the normal and defective specimens was the amplitude of the through-transmitted waveforms. The bandwidth was not an important factor for inspecting the PWR fuel rods. Therefore, detecting the presence of infiltrated water should be conducted by analyzing the variation in the transducer amplitude. This section may be divided by subheadings. It should provide a concise and precise description of the experimental results, their interpretation, as well as the experimental conclusions that can be drawn.

## 5. Concluding Remarks

In this study, a high-sensitivity piezo-electric ultrasonic transducer for detecting water infiltrated PWR fuel rods was designed and fabricated. The substance PMN-28%PT was used as the piezo-electric material due to its high piezo-electric constants. The through-transmitted waveforms were simulated using a newly developed KLM model. Moreover, the experimental setup was constructed using the same conditions used in the simulation. The through-transmitted waveforms of normal and defective specimens were obtained and compared with the simulation results. The results clearly demonstrated an amplitude difference between through-transmitted waveforms of normal and defective tubes, and this was the key feature for detecting infiltrated water in PWR fuel rods. However, the through-transmitted response waveform of normal PWR fuel rod was not fully analyzed because of the limitation of KLM model. To analyze the ultrasonic behavior of three-dimensional cylindrical structures with big acoustic differences, a finite element analysis was essential. In addition, the simultaneous measuring system for neutron dose and temperature was required for more harsh condition of various reactors. Therefore, these additional studies should be conducted for our future work. 

From the frequency spectrum analysis of the normal and defective waveforms, the bandwidth of the newly developed ultrasonic transducer was decreased because of its high piezo-electric coefficient. However, this was not a crucial factor in distinguishing the defective PWR fuel rods. In conclusion, the newly constructed KLM model shows good agreement with the experimental results and the newly developed PMN-28%PT-based ultrasonic transducer should be a promising device for use in NDE of components in NPPs.

## Figures and Tables

**Table 1:** Measurement conditions of X-ray diffractometry.

Parameters	Units	Values
X-ray source	·	Copper (Cu)
Load	kV	40
Sample mode	·	reflection
Scan mode	·	step scan
Scan range (2θ)	Degree, °	3600
Scan interval (2θ)	Degree, °	0.02
Monochromator	·	Curved graph
Detector	·	Scintillation Counter

**Table 2:** Measured material properties of PMN-28%PT single crystal.

Parameters	Units	Values
Density	Kg/m^3^	8000
Area of piezo-element	mm^2^	9
Dielectric constant at 1 kHz	$\varepsilon_{r}$	5400
Piezo-electric coefficient, *d*_33_	pC/N	1515
Longitudinal velocity	m/s	3600
Acoustic impedance	kg/m^2^s	29 × 10^6^
Operating frequency	MHz	5
Electro-mechanical coupling coefficient	$k_{33}$	0.9
Curie temperature	° C	160

**Table 3:** Simulation conditions of the KLM model.

Simulation Conditions	Units	Values
Area of PMN-PT	mm^2^	9
Resonance frequency of PMN-PT	MHz	5
Sound velocity of front matching layer	m/s	2800
Thickness of the test material	mm	14
Acoustic impedance of water		1.5
Acoustic impedance of front matching layer	kg/m^2^s	3.5 × 10^6^
Acoustic impedance of backing material		8 × 10^6^
Acoustic impedance of bonding material		3 × 10^6^

## Supplementary Materials

The following are available online at https://www.mdpi.com/1424-8220/19/12/2662/s1, Supplementary file 1: Matlab codes for through-transmission method using Matlab software, Supplementary file 2: raw data of excitation pulse.
