# Supplementary material for: Pb(Mg1/3Nb2/3)-PbTiO3-Based Ultrasonic Transducer for Detecting Infiltrated Water in Pressurized Water Reactor Fuel Rods
Source: Sensors (Basel). 2019 Jun 13;19(12):2662. doi: 10.3390/s19122662 (PMC6630703; doi:10.3390/s19122662)
Supplement: Supplementary file 1 [file sensors-19-02662-s001.zip › supplementary materials/Supplementary file 1.pdf]

1. Constructed the KLM model based through-transmission model

```
close all

%%%%%%%%%%%%%%%%%%%%%%%%%%%%%%%%%%%%%%%%%%%%%%%%%%%%%%%%%%%%%%%%%%%%%%%%  Input axis setting
%%%%%%%%%%%%%%%%%%%%%%%%%%%%%%%%%%%%%%%%%%%%%%%%%%%%%%%%%%%%%%%%%%%%%%%%

N = 2^12;                    % Sampling Number
t = linspace(0, 8, N);      % Sampling Time          [us]
dt = t(2) - t(1);            % Time step              [T/N]
f = linspace(eps, 1/dt, N);    % Sampling Frequency     [MHz] ∴ Fs = N/t

% eps = 2.220446049250313e-16

%%%%%%%%%%%%%%%%%%%%%%%%%%%%%%%%%%%%%%%%%%%%%%%%%%%%%%%%%%%%%%%%%%%%%%%%    Transfer Function
%%%%%%%%%%%%%%%%%%%%%%%%%%%%%%%%%%%%%%%%%%%%%%%%%%%%%%%%%%%%%%%%%%%%%%%%

w=f*(2*pi)*1e6;            % Sampling Frequency [rad/s] (Synchronized)
w0_in=3e6*2*pi;          % input  frequency Resonance frequency [rad/s]
ww_in=w/w0_in;

w0_out=1.3e6*2*pi;          % output  frequency
ww_out=w/w0_out;

Zt=45e6;          % load impedance
ohm=50;             % Impedance Matching

%%%%%%%%%%%%%%%%%%%%%%%%%%%%%%%%%%%%%%%%%%%%%%%%%%%%%%%%%%%%  N1
%%%%%%%%%%%%%%%%%%%%%%%%%%%%%%%%%%%%%%%%%%%%%%%%%%%%%%%%%%%%%%%%%%%%%%%%%%%%%%%%%%%%%%%%%%%%%%%%%%%%%%%%%%%%%

n=2;              % turn relationship

% electric matching matrix - turn relationship n
N1_11=n*ones(1, length(w));
N1_12=0*ones(1, length(w));
N1_21=0*ones(1, length(w));
N1_22=1/n*ones(1, length(w));

N1_in = cell2mat({N1_11; N1_12; N1_21; N1_22});
N1_out = cell2mat({N1_11; N1_12; N1_21; N1_22});

%%%%%%%%%%%%%%%%%%%%%%%%%%%%%%%%%%%%%%%%%%%%%%%%%%%%%%%%%%%%%%%%%%%%    N2
%%%%%%%%%%%%%%%%%%%%%%%%%%%%%%%%%%%%%%%%%%%%%%%%%%%%%%%%%%%%%%%%%%%%%%%%%%%%%%%%%%%%%%%%%%%%%

c0=3600e-12;           % Capacitance
L_in=1/(w0_in^2*c0);      % Serial Inductance
L_out=1/(w0_out^2*c0);      % Serial Inductance

% electric matching matrix - Serial Inductance
N2_11=1*ones(1, length(w));
N2_12=(-i*w*L_in);
N2_21=0*ones(1, length(w));
N2_22=1*ones(1, length(w));

N2_in = cell2mat({N2_11; N2_12; N2_21; N2_22});

N2_11=1*ones(1, length(w));
N2_12=(-i*w*L_out);
N2_21=0*ones(1, length(w));
N2_22=1*ones(1, length(w));

N2_out = cell2mat({N2_11; N2_12; N2_21; N2_22});
```

```
%%%%%%%%%%%%%%%%%%%%%%%%%%%  N3
%%%%%%%%%%%%%%%%%%%%%%%%%%%%%

kt=0.5;              % 전기-기계결합상수

cc_in=-c0./(kt^2*(sin(pi*ww_in)./(pi*ww_in)));              % C`_in
cc_out=-c0./(kt^2*(sin(pi*ww_out)./(pi*ww_out)));          % C`_out

% Matrix that characterizes capacitances C and C`
N3_11=1*ones(1, length(w));
N3_12=(i*(1./(w*c0)+1./(w.*cc_in)));
N3_21=0*ones(1, length(w));
N3_22=1*ones(1, length(w));

N3_in = cell2mat({N3_11; N3_12; N3_21; N3_22});

% Matrix that characterizes capacitances C and C`
N3_11=1*ones(1, length(w));
N3_12=(i*(1./(w*c0)+1./(w.*cc_out)));
N3_21=0*ones(1, length(w));
N3_22=1*ones(1, length(w));

N3_out = cell2mat({N3_11; N3_12; N3_21; N3_22});

%%%%%%%%%%%%%%%%%%%%%%%%%%%%    N4
%%%%%%%%%%%%%%%%%%%%%%%%%%%%%

zc=34e6;             % piezelectric impedance
tr_in=kt*(pi/(w0_in*c0*zc)^(1/2))*sin(pi*ww_in/2)./(pi*ww_in/2);          % Ψ
tr_out=kt*(pi/(w0_out*c0*zc)^(1/2))*sin(pi*ww_out/2)./(pi*ww_out/2);          % Ψ

% Matrix of the elements of the transformer Ψ
N4_11=1./tr_in;
N4_12=0*ones(1, length(w));
N4_21=0*ones(1, length(w));
N4_22=tr_in;

N4_in = cell2mat({N4_11; N4_12; N4_21; N4_22});

% Matrix of the elements of the transformer Ψ
N4_11=1./tr_out;
N4_12=0*ones(1, length(w));
N4_21=0*ones(1, length(w));
N4_22=tr_out;

N4_out = cell2mat({N4_11; N4_12; N4_21; N4_22});

%%%%%%%%%%%%%%%%%%%%%%%%%%  N5
%%%%%%%%%%%%%%%%%%%%%%%%%%%%%%%%%

zb=34e6;              % backing impedance

zz_in=pi*ww_in/2;                                                    % pτ
zib_in=zc*((zb+i*zc*tan(zz_in))./(zc+i*zb*tan(zz_in)));   % ZL : net impedance

zz_out=pi*ww_out/2;                                                    % pτ
zib_out=zc*((zb+i*zc*tan(zz_out))./(zc+i*zb*tan(zz_out)));   % ZL : net impedance

% Matching matrix between the backing and the piezoceramic
N5_11=1*ones(1, length(w));
N5_12=0*ones(1, length(w));
N5_21=-1./zib_in;
N5_22=1*ones(1, length(w));

N5_in = cell2mat({N5_11; N5_12; N5_21; N5_22});
```

```
% Matching matrix between the backing and the piezoceramic
N5_11=1*ones(1, length(w));
N5_12=0*ones(1, length(w));
N5_21=-1./zib_out;
N5_22=1*ones(1, length(w));

N5_out = cell2mat({N5_11; N5_12; N5_21; N5_22});

%%%%%%%%%%%%%%%%%%%%%  N6
%%%%%%%%%%%%%%%%%%%%%%%%%%%%%%%%%%

% Piezoceramic matrix connected to the backing
N6_11=cos(zz_in);
N6_12=(-i*zc*sin(zz_in));
N6_21=(-i*(1/zc)*sin(zz_in));
N6_22=cos(zz_in);

N6_in = cell2mat({N6_11; N6_12; N6_21; N6_22});

% Piezoceramic matrix connected to the backing
N6_11=cos(zz_out);
N6_12=(-i*zc*sin(zz_out));
N6_21=(-i*(1/zc)*sin(zz_out));
N6_22=cos(zz_out);

N6_out = cell2mat({N6_11; N6_12; N6_21; N6_22});

%%%%%%%%%%%%%%%%%%%%%   N7
%%%%%%%%%%%%%%%%%%%%%%%%%%%%%%%%%%%%%%

z1=2.74e6;          % epoxy impedance
l1=0.000001;        % epoxy 두께
v1=2800;             % velocity

% Piezoceramic matrix connected to the load through layers.(epoxy)
zz1=l1*w/v1;                              % pτ(epoxy)

N7_11=cos(zz1);
N7_12=(-i*z1*sin(zz1));
N7_21=(-i*(1/z1)*sin(zz1));
N7_22=cos(zz1);

N7_in = cell2mat({N7_11; N7_12; N7_21; N7_22});

N7_11=cos(zz1);
N7_12=(-i*z1*sin(zz1));
N7_21=(-i*(1/z1)*sin(zz1));
N7_22=cos(zz1);

N7_out = cell2mat({N7_11; N7_12; N7_21; N7_22});

%%%%%%%%%%%%%%%%%%%%%   N8
%%%%%%%%%%%%%%%%%%%%%%%%%%%%%%%%%%%%%%

zf_in=41.34e6;          %  input wear plate impedance
lf_in=0.7e-3;           % input wear plate 두께
vf_in=10600;            % input velocity

% Matrix of the 1st matching layer.(Front Matrial)
zzf_in=lf_in*w/vf_in;                               % pτ(Front Mat)

zf_out=41.34e6;          % output wear plate impedance
lf_out=1.5e-3;           % output wear plate 두께
vf_out=10600;            % output velocity
```

```
% Matrix of the 1st matching layer.(Front Matrial)
zzf_out=lf_out*w/vf_out;                                % pτ(Front Mat)

N8_11=cos(zzf_in);                  %   input wear plate matrix
N8_12=(-i*zf_in*sin(zzf_in));
N8_21=(-i*(1/zf_in)*sin(zzf_in));
N8_22=cos(zzf_in);

N8_in = cell2mat({N8_11; N8_12; N8_21; N8_22});

N8_11=cos(zzf_out);             %   output wear plate matrix
N8_12=(-i*zf_in*sin(zzf_out));
N8_21=(-i*(1/zf_in)*sin(zzf_out));
N8_22=cos(zzf_out);

N8_out = cell2mat({N8_11; N8_12; N8_21; N8_22});

%%%%%%%%%%%%%%%%%%%%%    N9
%%%%%%%%%%%%%%%%%%%%%%%%%%%%%%%%%%%%%%

z2=1.5e6;            % couplalnt
l2=0.000001;         % 두께
v2=1500;              % velocity

% Matrix of the 2nd matching layer.(Couplant)
zz2=-l2*w/v2;                           % pτ(Couplant)

N9_11=cos(zz2);
N9_12=(-i*z2*sin(zz2));
N9_21=(-i*(1/z2)*sin(zz2));
N9_22=cos(zz2);

N9_in = cell2mat({N9_11; N9_12; N9_21; N9_22});

N9_11=cos(zz2);
N9_12=(-i*z2*sin(zz2));
N9_21=(-i*(1/z2)*sin(zz2));
N9_22=cos(zz2);

N9_out = cell2mat({N9_11; N9_12; N9_21; N9_22});

%%%%%%%%%%%%%%%%%%%%%    N10
%%%%%%%%%%%%%%%%%%%%%%%%%%%%%%%%%%%%%%

zm=45.74e6;           % metal impedance
lm=30e-3;              % 두께
vm=5900;               % velocity

% Matrix of the metal matching layer.
zzm=lm*w/vm;                            % pτ(metal)

N10_11=cos(zzm);
N10_12=(-i*zm*sin(zzm));
N10_21=(-i*(1/zm)*sin(zzm));
N10_22=cos(zzm);

N10 = cell2mat({N10_11; N10_12; N10_21; N10_22});

%%%%%%%%%%%%%%%%%%%%%%%%%%%%%%%%%%%%%%%%%%%%%%%%%%
%%%%%%%%%%%

% Transfer Matrix.(Nt = )
Nt = cell2mat({ones(1, length(w)); zeros(1, length(w)); zeros(1, length(w)); ones(1,
length(w))});
```

```
 Nt = Matrices_Product(Nt,N1_out);
 Nt = Matrices_Product(Nt,N2_out);
 Nt = Matrices_Product(Nt,N3_out);
 Nt = Matrices_Product(Nt,N4_out);
 Nt = Matrices_Product(Nt,N5_out);
 Nt = Matrices_Product(Nt,N6_out);
 Nt = Matrices_Product(Nt,N7_out);
 Nt = Matrices_Product(Nt,N8_out);
 Nt = Matrices_Product(Nt,N9_out);
 Nt = Matrices_Product(Nt,N10);
 Nt = Matrices_Product(Nt,N9_in);
 Nt = Matrices_Product(Nt,N8_in);
 Nt = Matrices_Product(Nt,N7_in);
 Nt = Matrices_Product(Nt,N6_in);
 Nt = Matrices_Product(Nt,N5_in);
 Nt = Matrices_Product(Nt,N4_in);
 Nt = Matrices_Product(Nt,N3_in);
 Nt = Matrices_Product(Nt,N2_in);
 Nt = Matrices_Product(Nt,N1_in);

%%%%%%%%%%%%%%%%%%%%  Eletric Supply impedance Ze
%%%%%%%%%%%%%%%%%%%%

zg=(zc^(1/2))*(Zt^(1/2));
Rm=((4*kt^2)/(pi*w0_in*c0))*(zc/(zg+zb)).*((w0_in./(w)).^2).*(sin(pi*ww_in/2)).^2;
Xe=(ohm/w0_in*c0)*(2-kt^2-(2*kt^2)*((2*zc^2-zg^2-zb^2)/(zg+zb)^2));

Ze=Rm+i*Xe;

%%%%%%%%%%%%%%%%%%%%%%%%%%%  transfer function
%%%%%%%%%%%%%%%%%%%%%%%%%

HT=2*Zt./(-Zt*Ze.*Nt(3,:)+Nt(1,:).*Ze-Nt(2,:)+Nt(4,:)*Zt);
%HT=2*Ze./(-Ze.*Ze.*Nt(3,:)+Nt(1,:).*Ze-Nt(2,:)+Nt(4,:).*Ze); % 1차 수정

ss=(Nt(3,:).*Ze*Zt-Nt(1,:).*Ze-Nt(4,:)*Zt+Nt(2,:)).^2;

%ss=(Nt(3,:).*Ze.*Ze-Nt(1,:).*Ze-Nt(4,:).*Ze+Nt(2,:)).^2;

HR=4*Ze*Zt./ss;                    % HR(w) - Frequency domain

HR_l=20*log(max(HR)./HR);

%%%%%%%%%%%%%%%%%%%%%%%%%%%%%   spike Input
%%%%%%%%%%%%%%%%%%%%%%%%%%%%%

% y = pulse_ref(0.35, 2.2, 2.8, t);  % Time domain [us]/ 볼트, 주파수(MHz), 대역폭,
% y = -exp(-(t-4).^2/2).*sin(w0.*t*1e-6);
% y = transpose(Importfile('Input_Signal.xlsx','Gaussian_1',2,4097));

V0=0.33;                             % input voltage (V)
t0=0.1;

a1=0.2;
a2=5000;

y=pulser(t0,a1,a2,V0,t);

yf1 = FourierT(y, dt);              % Frequency domain [MHz]
yt = IFourierT(yf1, dt);

%%%%%%%%%%%%%%%%%%%%%%%%%%%%%   Output Signal
%%%%%%%%%%%%%%%%%%%%%%%%%%%%%%
```

```
Vout = yf1.*HT;                  % Vin(f)、HR(w) - Frequency domain
Vout5 = Vout.*(f < 1/dt/2);

Vout_t = IFourierT(Vout5, dt);    % Vout(t) - Time domain

%%%%%%%%%%%%%%%%%%%%%%%%%%%%%      Plot
%%%%%%%%%%%%%%%%%%%%%%%%%%%%%%%%%%%%%%

subplot(2,3,1);plot(f(1:1000),abs(yf1(1:1000)));   %% vin fre
subplot(2,3,1);ylabel('|Vin|');
subplot(2,3,1);xlabel('Frequency[MHz]');

subplot(2,3,2);plot(f(1:100),0.1*abs(HT(1:100)));     %% ht fre
subplot(2,3,2);ylabel('|HT|');
subplot(2,3,2);xlabel('Frequency[MHz]');

subplot(2,3,3);plot(f(1:50), abs(Vout(1:50)));     %% vout fre
subplot(2,3,3);ylabel('|Vout|');
subplot(2,3,3);xlabel('Frequency[MHz]');

subplot(2,3,4);plot(t, y);                        %% vin time
subplot(2,3,4);ylabel('|Vin|');
subplot(2,3,4);xlabel('time[us]');

subplot(2,3,5);plot(t, 2*real(Vout_t)*0.7e4);
subplot(2,3,5);
subplot(2,3,5);

subplot(2,3,6);plot(t(1:3000), 2*1e4*real(Vout_t(1:3000))*1e-3); %% time vout
subplot(2,3,6);ylabel('|Vout|');
subplot(2,3,6);xlabel('time[us]');

Voutt = 7*transpose(2*real(Vout_t));
```
